# Low-Temperature Plasma Techniques in Biomedical Applications and Therapeutics: An Overview

**DOI:** 10.3390/ijms25010524

**Published:** 2023-12-30

**Authors:** Chandrima Karthik, Sarath Chand Sarngadharan, Vinoy Thomas

**Affiliations:** 1 Department of Materials & Mechanical Engineering, University of Alabama at Birmingham, 1150 10th Avenue South, Birmingham, AL 35205, USA; ckarthik@uab.edu; 2 Georgia Institute of Technology, 901 Atlantic Dr., Atlanta, GA 30332, USA; sarathchands@gmail.com

**Keywords:** Low-Temperature Plasma (LTP), plasma medicine, biomaterials

## Abstract

Plasma, the fourth fundamental state of matter, comprises charged species and electrons, and it is a fascinating medium that is spread over the entire visible universe. In addition to that, plasma can be generated artificially under appropriate laboratory techniques. Artificially generated thermal or hot plasma has applications in heavy and electronic industries; however, the non-thermal (cold atmospheric or low temperature) plasma finds its applications mainly in biomedicals and therapeutics. One of the important characteristics of LTP is that the constituent particles in the plasma stream can often maintain an overall temperature of nearly room temperature, even though the thermal parameters of the free electrons go up to 1 to 10 keV. The presence of reactive chemical species at ambient temperature and atmospheric pressure makes LTP a bio-tolerant tool in biomedical applications with many advantages over conventional techniques. This review presents some of the important biomedical applications of cold-atmospheric plasma (CAP) or low-temperature plasma (LTP) in modern medicine, showcasing its effect in antimicrobial therapy, cancer treatment, drug/gene delivery, tissue engineering, implant modifications, interaction with biomolecules, etc., and overviews some present challenges in the field of plasma medicine.

## 1. Introduction

Plasma is a combination of charged particles with ions and electrons. There have been many applications for plasma technology since the dawn of the 20th century. Fluorescent lighting is one of the first known commercial uses of plasma technology [1,2]. The state of plasma is not a mere combination of charged species, although it can be broadly defined as a quasineutral gas of charged and neutral species that exhibits a collective behavior of localized concentrations of positive and negative charges by long-range Coulomb forces [3,4]. Two types of plasmas can be artificially generated by changing the physical properties of a neutral gas, (1) thermal plasma or hot/high-temperature/equilibrium plasma and (2) non-thermal plasma (NTP) or cold-atmospheric plasma (CAP) or low-temperature plasma (LTP) non-equilibrium plasma. Thermal plasma, for example, solar plasma, nuclear plasma, laser fusion plasma, etc., is characterized by high energy density and possesses a thermal equilibrium between the associated particle ions and electrons. Thermal plasma has applications in electronics and heavy industries for cutting, welding, spraying, metallurgy, etc., [5]. However, the non-thermal plasma is characterized by low energy density and a thermal non-equilibrium between the electrons and the constituent particles [6]. One of the main features of LTP is the incredibly large differences in temperature of its constituent species. The heavier gas species and ions often possess temperatures near room temperature, whereas the thermal parameter of free electrons goes up to 50,000 K [7]. Such a high electron temperature can break down the chemical bonds in neutral gas molecules and generate reactive chemical species. This particular ability of LTP has been utilized for the degradation of hazardous chemicals/pollutants and manipulation (catalysis) of chemical reactions [8]. As stated above, the average temperature of LTP stays around room temperature, and it was selectively generated and used in biomedical research [9], therapeutics [10], dentistry [11], agriculture [12], food decontamination [13], electrical device fabrications [14], surface modifications/coatings, and fine tunings in material chemistry applications [15], etc.

Low-temperature plasma-assisted procedures are the core of plasma medicine, which is a relatively new field of study in modern medicine. Since the LTP technology is administered at a low temperature, it can be used to treat a variety of biological materials, comprising solids, liquids, and aerosols [16]. The last two decades have witnessed enormous developments in the practical applications of LTP in biology and medicine due to its non-thermal behavior and the abundance of reactive oxygen and nitrogen species (RONS) in it [17,18]. The translational biomedical plasma application ranges from sterilization to wound healing, antimicrobial treatment, dermatology, dentistry, surgical procedures, oncology, etc. The ability of non-thermal plasma to trigger biological responses in cells/tissues can play a vital role in modern medicine. For example, plasma medicine preferentially targets cancer cells because their mechanistic activities typically depend on the production of reactive species. The key active agents accountable for the biological effects of direct and indirect plasma treatments have been identified as reactive oxygen species (ROS) such as hydroxy (OH), hydroxy radical (^•^OH), superoxide (O_2_^−^•), hydrogen peroxide (H_2_O_2_), ozone (O_3_), and singlet oxygen (^1^O_2_), and reactive nitrogen species (RNS) such as nitric oxide (•NO), nitrogen trioxide (N_3_O), nitrogen dioxide (NO_2_), dinitrogen tetroxide (N_2_O_4_), nitrous oxide (N_2_O), and peroxy nitrate (ONOO^−^), and they may offer a promising new approach to the therapy of tumors [17,19]. The existence of extremely active, transient ROS generated during ionization provides a special chemical opportunity to modify target cell responses, which is advantageous for direct plasma therapies. Understanding the fundamental mechanisms of action of plasma is crucial for its ability to elicit the desired impact in the intended tissue, which ultimately determines the efficacy of these therapies. LTP-based transdermal drug delivery [20] and gene delivery systems are also revolutionary strategies in plasma medicine. The molecular and physiological processes that control protein adsorption, cell adhesion, and cell proliferation can be specifically controlled by plasma-manipulated nanoengineered surfaces. Improved implant surface functionalization for better cell adhesion and proliferation can also be created by various plasma treatments. This review is focused on various applications of LTP in medicine and biomedical applications.

## 2. Introduction Low-Temperature Plasma on Medicine

### 2.1. Low-Temperature Plasma Pathogen Inactivation and Antimicrobial Therapy

The COVID-19 pandemic outbreak made the entire globe aware of how devastating the microbes can be. One of the major issues that has to be addressed is the increase in multidrug-resistant pathogens. According to the latest report published by the World Health Organization, multidrug-resistant bacteria is considered as one of the top 10 global public health risks [21]. LTP technology is one of the emerging technologies that could be essential in the near future and supports the effort to combat multidrug-resistant pathogens and biofilms. Several vital antimicrobial techniques, from materials-based to physics-based biological decontamination methods, have been made possible by cold plasma. These techniques can be applied to both prevention and post-infection management for multi-level infection control [22]. The reactive oxygen and nitrogen species (RONS) from LTP are essential for the inactivation of microorganisms [4,23]. LTP–antimicrobial efficacy is highly influenced by various factors such as input power, operating gas, flow rate, exposure distance, exposure time, etc. 

**Figure 1:**
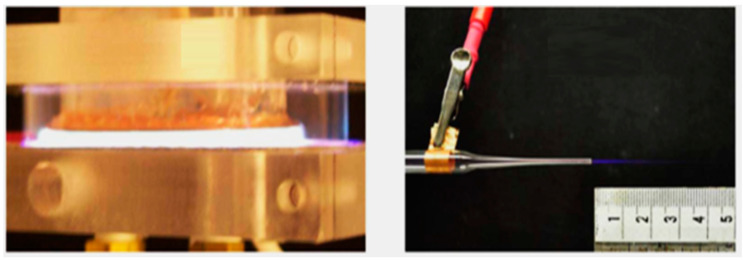
Two sources of LTP: dielectric barrier discharge (DBD) in argon driven by repetitive short duration (ns–µs) high-voltage pulses (**left**); a micro-jet using helium as operating gas, generating a cold plasma plume about 2.5 cm in length (**right**). Reproduced from Laroussi M, Cold plasma in medicine and healthcare: The new frontier in low temperature plasma applications. Front. Phys. 8:74. doi:10.3389/fphy.2020.00074 under the terms of the Creative Commons Attribution License (CC BY) [2].

LTP can be created in many ways including a simple gas discharge, microwave discharge, rf radio frequency (rf) discharge, corona discharge, gliding arc discharge, glow discharge, dielectric barrier discharge (DBD), etc., [24]. According to how they are built in relation to the treated object, LTP methods can be broadly categorized into three types: direct LTP, indirect LTP, and hybrid LTP [25,26,27]. The treated object is considered one of the electrodes in direct LTP. DBD is one of the most common direct discharge devices that generate a large volume of non-equilibrium plasma for clinical applications. It is well known for its efficiency in maintaining homogeneous discharge at atmospheric pressure and the power to generate a significant amount of chemically reactive species [28]. In addition to that, a more bio-tolerant non-equilibrium atmospheric pressure plasma jet (N-APPJ) device is also utilized to emit plasma plums especially to biological cells and tissues without damaging their physiology [29]. N-APPJ is an example of an indirect plasma discharge device used in biomedical applications, mainly working based on convection and diffusion. Figure 1 represents a typical DBD (left) and an N-APPJ (right) device used as sources for LTP. The hybrid LTP combines the aforementioned two types having a grounded mesh-type electrode [30,31]. A variation in antibacterial effect has been noted in several investigations comparing direct and indirect methods. Direct LTP therapy showed a larger inactivation area than indirect LTP treatment [32]. The chemical characteristics and LTP discharge characteristics differ according to the type of gas. Using various operating gases or a combination of gases, numerous investigations have proven the antibacterial effectiveness of LTP [33]. In some instances, the antibacterial effect of gas movement was investigated, which does not affect the sample [34]. Sharma et al. [35] analyzed the influence of feeding gas flow rate in bacterial inactivation using Argon (Ar) and Helium (He) gas, and they noticed a decrease in the pathogen inactivation with the increased Ar gas flow; however, the observation was upturned while using He as the feeding gas [35]. The power and frequency have also a significant effect on pathogen inactivation; the inactivation efficiency of LTP increases with the increase in the input frequency and (or) power [36,37]. Studies with S. aureus show the bacterial inactivation efficiency of LTP by varying voltage. A change in voltage can also change the concentration of the reactive species present and thus alter the LTP antimicrobial efficiency [38]. Another important parameter having significant effects on pathogen inactivation is the LTP exposure time and its distance from the sample specimen. The increased distance between the source and the sample will decrease the efficiency [39]. The exposure time also plays a major role in the efficiency of the LTP treatment and may vary depending on the nature of the microorganism [40,41]. The plasma irradiation studies conducted in *E. coli* and *P. aeruginosa* show that exposure time is directly proportional to the inactivation efficiency [39,42].

LTP-based antimicrobial treatment is becoming popularized due to its easiness and effectiveness. Microbial infection keratitis is the main reason for corneal inflammation and ulceration. It is brought on by the growth and invasion of bacteria, fungi, and viruses in the cornea. Martines et al. [43] reported that a chemically enriched afterglow-based plasma device can be detrimental to these pathogens, and the number of these pathogens decreases within a few minutes of treatment without altering the corneal morphology [43,44]. In vitro dental pathogens studies carried out using cold atmospheric plasma and various database searches showed that LTP is a viable alternative for antibacterial therapy with a quick clinical implementation [45]. LTP can be used in various agriculture sectors for different microbial interventions on crops as well [46]. Low-temperature gas plasmas are good for microgravity biofilms-based applications. New methods of stopping biofilm formation and disrupting biofilms are required since microgravity biofilms interfere with both current and future space missions [47]. LTP could be used to decontaminate implants [48,49], to destroy genetically modified bacteria [50], for various hygiene applications [51,52], and in natural fabrics [53].

Bacterial biofilms are a greater threat in the biomedical and food industries. There is an urgent need for new therapeutic approaches that can eliminate bacteria in biofilms with little to no danger of triggering resistance. The need for new and complementary antimicrobial strategies is driven by the widespread and increasing resistance to broad-spectrum antibiotics as well as concerns about the health effects of numerous biocidal agents. Increased mechanical and antibacterial stability is a property of biofilms, which have been the focus of much investigation [54]. LTP was utilized to reduce *Enterococcus faecalis* developing as biofilms by >99.9%, which is comparable to the antibacterial activity of 0.2% chlorhexidine. The fact that LTP performs fully without any interaction is what makes it excellent [55]. 

In a variety of biotechnology applications, plasma-activated water (PAW), a by-product of a non-thermal plasma reaction with water that contains a wide range of highly reactive oxygen and nitrogen species, offers a sustainable alternative to conventional methods for removing microorganisms from surfaces [56]. Water undergoes several intricate reactions, including excitation, dissociation, and ionization, when the high-energy electrons produced by a plasma discharge collide with it. In addition to the creation of many active particles [57], LTP causes several changes in the physical properties of water, such as viscosity, surface tension, contact angle, etc. A recent study from Prof. Fridmas’s lab reports that the physical properties of water change with plasma activation. One of the important observations is that the percentage of changes that occurred in the plasma-activated water is greater than the percentage of the added plasma species, and the entire solution showed a non-ideal behavior after the treatment. The plasma treatment lowered the surface tension of the water by 6.1% and inclined the viscosity by 12.8% at room temperature. There was a dramatic change in the contact angle; a 36% reduction was observed with a glass surface. All these changes in the physical properties of PAW indicate that the plasma treatment can alter the crystalline behavior of water to an amorphous nature even at lower temperatures [58]. PAW has several advantages due to its antimicrobial efficiency; many recent reports demonstrated PAW as a biocidal agent for biofilm elimination without bacterial resistance. Gram-positive bacteria are more resistant to plasma than Gram-negative due to the difference in the peptidoglycan structure (far thicker layer) and level of acetylation [59]. The studies of PAW-bacterial bio-film interaction were recently reviewed by Mai-Prochnow et al. [60] and have provided an overall schematic representation of the mechanisms of how the ROS and RNS disrupt the biofilm structure [60]. 

Plasma-activated liquids (PAL) are also a very effective medium for disinfecting oral bacteria. According to Hong et al. [61], PALs were unable to eliminate all the microbes or stop them from regrowing. The PALs were not harmful to mammalian cells. Hydrogen peroxide content in the liquids could be raised by plasma treatment but not to a level that would entirely render all microbes inactive [61]. “Frugal innovation” or “frugal engineering” refers to the idea of making technology more basic and without “frills” to make it less expensive and possibly more durable while addressing crucial social and environmental needs [62]. The concept of frugal innovation readily matches air plasma technology. Cheap, portable solar energy panels can be used to power frugal plasma sources. When used in conjunction with water, a cheap portable air plasma “corona pen” can be extremely effective in killing bacteria and even inactivating biofilms resistant to antibiotics [63,64]. A wound that has germs may take longer to heal and require more time in the hospital. Patients with burns are particularly vulnerable to infections caused by *Pseudomonas aeruginosa* and *Staphylococcus aureus* [65]. With various degrees of efficacy, LTP devices—primarily plasma jets—have been used on chronic wounds to lower bacterial concentration and promote in vivo wound healing [66]. LTP with pulsed electric fields (PEFs) demonstrated considerable synergistic actions for the inactivation of germs compared to separate treatments [52,67].

### 2.2. Cancer Treatment with Low-Temperature Plasma

**Figure 2:**
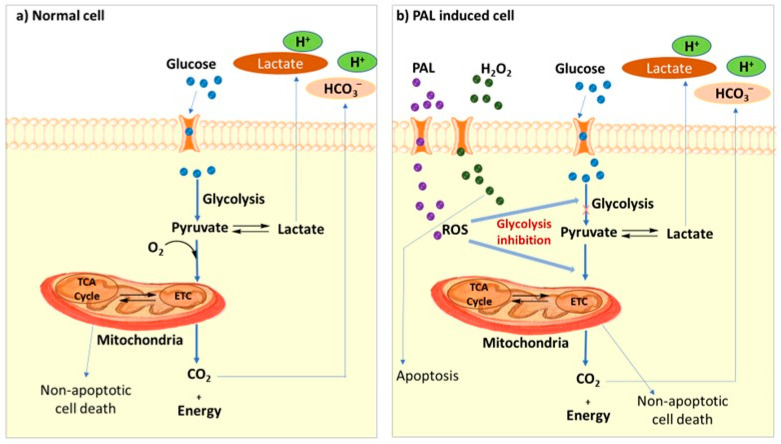
Pathways for inhibition of glycolysis and tricarboxylic acid (TCA) cycle and electron transfer chain (ETC) by plasma-activated Ringer’s lactate solution (**a**). Extracellular H_2_O_2_ induces apoptosis and non-H_2_O_2_ PAL components induces non-apoptotic cell death (**b**) [76,85].

Ever since the pioneering works of Friedman and Keider early this century, LTP-based cancer therapy has been established as a promising therapeutic technique in the field of cancer medicine, especially as a tool for the selective apoptosis of cells and tissues. The area of plasma medicine has then started developing through in vitro studies on different cancer cells [68]. A detailed analysis of plasma-based cancer treatment has been reviewed very recently by Alizadeh et al. [69] with a special focus on its clinical translation into modern therapeutics [69]. The use of plasma in cancer treatment might be either direct or indirect. Specially designed plasma devices, for example, gas plasma jets/pens can be used as a surgical tool to treat cancer cells externally and come under the category of direct plasma treatment [70]. On the other hand, LTP-activated solution-based therapy is comparatively new and is an indirect therapeutic approach to treating some inaccessible or inoperable tumors. Water, saline, cell culture medium, Ringer’s lactate solutions (RL), etc. are a few examples of the liquids used to prepare LTP-activated solutions. These solutions can be easily activated by dielectric barrier discharge plasma devices such as gas plasma pens or atmospheric pressure plasma jets. Some of the recent reports underlined that the plasma-activated solutions (indirect treatment) show better selectivity to induce apoptosis in cancer cells with minimal damage to the healthy cells, which is contrary to the direct plasma treatment that may result in considerable cytotoxicity to the normal cells. Tanaka et al. [71] examined the inhibitory effect of plasma-activated Ringer’s lactate in the cellular respiratory system of HeLa cells and outlined a novel plausible mechanism for PAL-induced apoptosis (Figure 2) [71,72,73,74,75,76]. These discrepancies in the results show that not only the long-lived reactive species are responsible for the anti-cancer activity of plasma, but in addition, the short-lived species and other physical factors in plasma can also impart cell toxicity in living cells. The major difference between direct and indirect plasma treatment is the lifetime of the reactive species; direct plasma therapy enables providing short-living species such as superoxides (O_2_) and hydroxyl radicals (OH^•^); however, in the indirect process, longer-lived species such as NO_2_, H_2_O_2_, ONOO^−^, NO_3_, etc. would be the reactive species [77]. Many recent reports demonstrated the ability of these reactive nitrogen and oxygen species (RNOS) to the selective apoptosis of cancer cells and its mechanisms of action [78,79,80,81]. This idea is similar to photodynamic therapy (PDT), which is already used in clinical oncology for a variety of cancer treatments [82]. The mechanism for the interaction of a living cell with plasma is highly complex and depends on the dose of plasma, cell size/type, biochemical differences in the cell membrane, metabolic rates, cell cycle differences, etc. The intermediate doses of plasma can selectively interact with the cells which show relatively high metabolic rates and effectively control cell death without necrosis [83,84]. 

**Figure 3:**
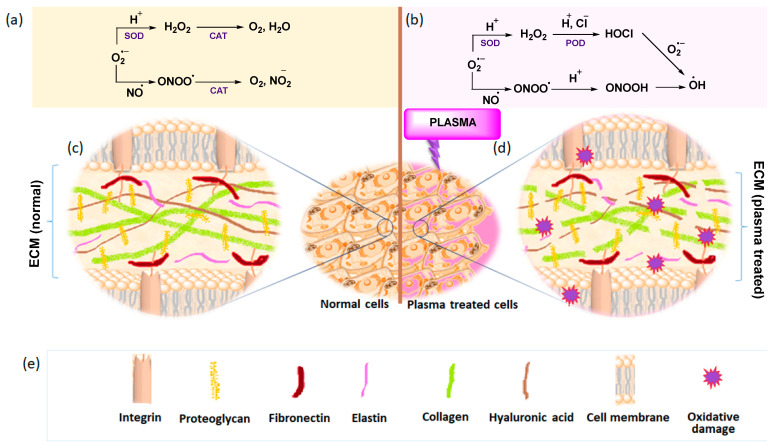
(**a**) Catalase (CAT) and superoxidase dismutase (SOD) catalyzed cell signaling pathways in normal (cancer) cells, (**b**) HOCl and ONOO^−^ pathway in plasma-treated cells, (**c**) representation of an extracellular matrix of normal (cancer) cells (**d**) plasma-treated ECM under oxidative stress, (**e**) components of extracellular matrix. Reproduced from Privat-Maldonado, et al., Modifying the tumor microenvironment: Challenges and future perspectives for anticancer plasma treatments. Cancers 2019, 11, 1920 [87] under the terms of the Creative Commons Attribution License (CC BY).

It is well established that cancer development is associated with physiological changes in the cellular and non-cellular components or extracellular matrices (ECMs), which is collectively called the ‘tumor microenvironment’ (TME) [86]. LTP therapy is a novel anti-cancer approach that targets the TME components at a further distance by triggering specific signaling pathways and activating the immune response. Due to the excess intracellular RONS level, cancer cells possess a higher rate of redox baseline and certainly must keep adequate antioxidant levels to prevent apoptosis. Any further increment in the intracellular reactive species of such RONS-enriched cells (application of plasma-induced external ROS) could induce oxidative damage to its ECM components and ultimately lead to selective apoptosis [87,88,89,90,91]. The mechanism of the plasma–cancer cell interactions can be one of two main types of cellular responses. First, the reactive species of plasma initiates and catalyzes the peroxidation of biomolecules in the cell membrane, which leads to cell detachment by the temporary loss of cell adhesion (permeabilization of the cell membrane) [92,93,94,95,96,97,98]. The second one is plasma-induced apoptosis, which occurs through intracellular biochemical changes (oxidative stress induced in the intracellular organelles) with controlled cell signaling pathways [99,100,101]. Even if the topic is still under debate, there are experimental shreds of evidence that the RONS, especially singlet oxygen (^1^O_2_) can interfere with the proliferation signaling pathways in the ECM and turn it into selective apoptosis signals [78,79,80,102]. There are two main apoptosis-inducing pathways associated with cell signaling, the HOCl pathway, and the ONOO^−^ pathways (these are the chemical species responsible for the formation of hydroxyl radical (^•^OH) in the vicinity of the cell membrane that leads to lipid peroxidation). In normal(cancer) cells, the enzyme ‘catalase’ suppresses both HOCl and ONOO^−^ pathways and restricts the generation of hydroxyl radicals. However, upon the induction of plasma to cancer cells, the enzyme ‘catalase’ will be selectively inactivated by singlet oxygen and leaves the other enzymes like superoxidase dismutases (SODs) and peroxidase (POD) to keep activating both the HOCl and ONOO^−^ pathways. The schematic representation of the plasma–cell interactions is detailed in Figure 3. 

In order to prove the interaction of LTP with cancer cells, Kaidar and co-workers used a cold plasma jet to induce atmospheric plasma into cancer cells both in vivo and in vitro. The results showed that the thermal effect associated with the plasma induction was negligible, and the plasma selectively ablates the cancer cells without causing any major damage to the healthy cells. It was also notable that the pH of the medium remains unchanged and has a minimal impact on the cellular bio-environment. With an in vivo study, they successfully endorsed the proof of principle: that a two-minute irradiation of plasma was effective in destroying mid-sized tumors in nude mice without any thermal damage [103]. 

As we know, the complete removal of cancer cells by surgery is challenging due to the local regional recurrence (LRR). In this scenario, plasma can be used as a combination therapy with surgery. The timely optimization of plasma during the course of treatment is a significant issue that needs to be tackled. The biological effects are influenced by RONS levels, which are directly impacted by plasma. Mumtaz et al. [104] assessed the plasma on-times of 25, 50, 75, and 100 ms in order to assess the electrical, optical, and soft jet features for two distinct duty ratios: 10% and 36%. By incorporating the optimization of plasma on-time to boost the soft plasma jet’s efficacy for biomedical applications, the study’s outcomes offer a noteworthy signal of development [104]. Canady Helios Cold Plasma (CHCP) is a comparatively new and safe LTP device that can selectively induce cancer cell death. The technology is still in the development stage and was approved by the FDA for a phase I clinical trial involving 20 stage IV cancer patients. The early results are promising, and it showed that this synergistic approach of surgery and plasma treatment significantly reduced the local regional recurrence of cancer cells [51,105]. Pulsed electron avalanche knife (PEAK) PlasmaBlade (PPB) is another LTP-based surgical tool that can improve the surgical outcome significantly more than the conventional approach. Although more shreds of evidence are needed, it has several advantages like shorter wound-healing time, pain-free swallowing, minimal collateral tissue damage, etc. [106,107]. Along with that, a recent clinical case study demonstrated the advantages of a mono-polar plasma knife in unilateral breast-conserving surgery over the traditional mastectomy. The monopolar low-temperature plasma scalpel expedites the surgical process minimizes blood loss during the procedure and enhances the quality of life for patients [108]. The area focused on the potential of LTP as an anti-cancer treatment and how well it works in conjunction with medication therapies has also shown noteworthy improvements these days. Chen et al. [109] investigated the combined synergistic effects of PAM (helium-atmospheric pressure plasma jet-activated RPMI medium) and Doxo treatment led to greater lethality in cancer cells compared to those treatments alone [109]. Very recently, Boeckmann et al. [110] studied the effect of plasma-treated small molecules in dermato-oncology via both in vitro and in vivo experiments to address the synergic mechanism of LTP to cancer cells [110]. They have optimized a library of 155 molecules, among which 6-Methyl-3-(2-fluorobenzoyl)c hromone (Sm837) has been selected for their experiments. Sm837 exhibits low cancer toxicity alone; however, its anti-cancer efficiency increases in combination with LTP treatment. In light of the need for new and creative approaches to treating various cancers and taking into account the benefits of combination therapies with regard to secondary therapy resistance, this area of research not only offers a promising combination of drugs in conjunction with cold gas plasma, but it also outlines a progression from an initial 3D screening method to in vitro validation and in ovo followed by in vivo confirmation. 

### 2.3. Plasma-Induced Changes in the Chemical Structure of Biomolecules

The capability of low-temperature plasma to disrupt the chemical bonds in complex molecules was utilized to improve the biological activities of natural products such as quercetin [111], naringin [112], the stilbenoid trans-resveratrol [113], etc. The plasma-treated flavonoid, quercetin showed increased α-glucosidase inhibitory activity and radical-scavenging activities compared to the natural quercetin. The results point out that the reactive species/free radicals might be able to hydrate and break the C-O bond in quercetin, which leads to low molecular phenolic compounds such as (±)-taxifolin, (±)-alphitonin, catechuic acid derivatives, etc., which is responsible for the improved biological activities. 

Similarly, a plasma-treated methanolic solution of *trans*-resveratrol (TR) showed enhanced inhibitory activities for α-glucosidase and α-amylase than its naturally occurring analog. The isolation and the structural characterization of the products from the plasma-induced TR showed the presence of six different species in an induction time of 40 min, out of which two of them are novel dimeric forms of TR connected by a methylene linkage (having the trivial names methylenebisresveratrol-A and methylenebisresveratrol-B) and four known molecules (−)-ε-viniferin, (+)-ε-viniferin, 3,5-dihydroxybenzaldehyde, and p-hydroxybenzaldehyde. The dimers obtained in the process exhibited a profound increase in both the α-glucosidase and α-amylase inhibition activities. 

A plasma-modified form of the natural flavonoid, naringin (a flavanone-7-O-glycoside between naringenin and the disaccharide neohesperidose, which occurs naturally in citrus fruits) was also well studied for its antioxidant activity, tyrosinase inhibition, and antimicrobial activities. The biological activities, especially the radical-scavenging ability of this plasma-induced naringin displayed a substantial increment in the antioxidant activities compared to its natural analog. It was reported that the concentration of the phenolic content of naringin rises from 172.50 to 225.83 ppm with the treatment of DBD plasma. Here, the mechanism of plasma interaction with naringin could be plausibly explained in such a way that the low-temperature plasma can chemically interact with the glycosidic bond in naringin and deglycosylated it into naringenin and the corresponding sugar molecules; these stable polyhydroxy molecules are responsible for the enhanced activities. 

It is also established that plasma-induced poly(oligo)merization has many advantages, and the most relevant one among them is that the reaction can be carried out under neat (no solvent involved) and low-temperature conditions [114]. The so-formed polymers have comparatively better physical characteristics like surface properties and shear strength. The application of atmospheric low-temperature plasma on macromolecules like lignocellulose can be used to improve the process of the biorefining of lignocellulose [115]. They reported that the plasma-induced chemical degradations in lignocellulose are mainly due to the successive bond cleavages of carbon–oxygen and carbon–carbon in its macromolecular skeleton and these structural changes were consistent with lignins of varying origins. Recently, Choi’s research group effectively utilized a non-thermal DBD plasma as a green and convenient method for the oligomerization of phloroglucinol through methylene linkages [116]. One such condensation product, a phloroglucinol-based pentamer, showed potential inhibitory activity against α-glucosidase. Based on this observation, the authors claimed that the DBD plasma is a green chemical methodology for the chemical modifications of drug-like molecules.

### 2.4. Low-Temperature Plasma-Assisted Drug/Gene Delivery Systems and Tissue Engineering

One of the important features of non-thermal plasma is that it can operate at biological temperatures. The biomedical applications of low-temperature plasma have been extended to gene transfer therapies and drug delivery. Several methods were developed for inducing foreign genes into the target cells, including gene transfection with genetically engineered viruses. Due to many drawbacks of these viral treatments, less virulent viruses were also invented for gene transfection procedures; however, it is comparatively less efficient than the viral vectors [117]. Physical methods like electroporation, microinjection, the application of calcium phosphate, and cationic lipids are also used for gene therapy, and these techniques are classified under the non-viral gene transfer processes [118]. However, these methods lack specificity due to their invasive nature to the healthy cells and tissues. In this scenario, the non-contact LTP treatment would be an effective alternative to gene therapy with reduced local discomfort from the exposure [119]. 

The pulsed electric field-based electrophoresis is one the most common methods used in gene transfection techniques, but the method fails to transfer genes to primary cells effectively [120]. In 2005, Ogawa et al. [120] presented an ion-based strategy, which is a plasma technique for transferring the gene to various types of cells without affecting the proper cell functioning. They successfully transferred green fluorescent protein (GFP) plasmid into post-miotic neuronal cells obtained from the cerebral cortex of rats using a low-temperature plasma surface treater. To prove the general applicability of the developed protocol, they investigated the effect of plasma in cells like HeLa and CHL (adherent cell lines), Jurkat cells (suspended cell line), and HUVEC (primary cells), and the results were affirmative with their perception [120]. Later, Connolly et al. [121,122,123] thoroughly investigated the applicability of this plasma technique for the delivery of DNA vaccines, fluorescent tracer molecules, etc. [121,122,123]. 

**Figure 4:**
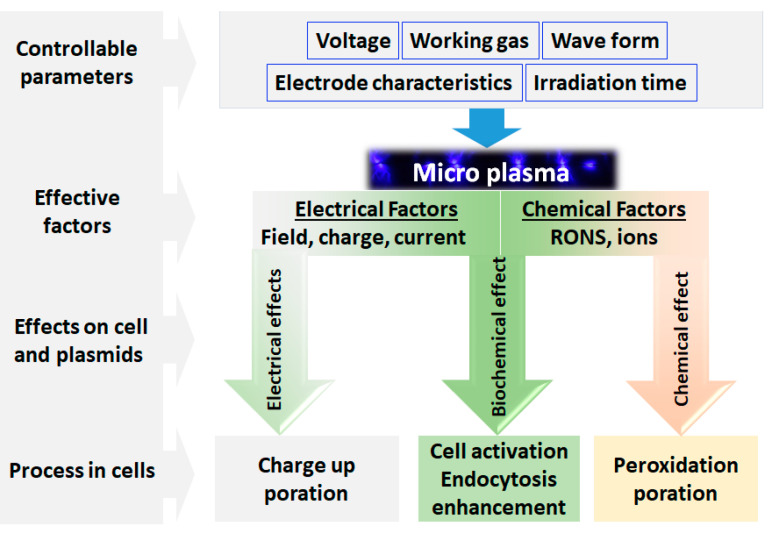
A schematic representation of the factors affecting the plasma gene transfection.

In 2016, Jinno’s research group studied the plasma-induced electrical and chemical factors in the process of gene transfection, which is a method of introducing foreign nucleic acids into eukaryotic cells in order to change the genetic composition of the host cell. They have used three experimental tools to analyze the effect of plasma induction: (1) a laser-produced plasma to investigate the chemical factors, (2) plasma irradiation on artificial cells like liposomes to evaluate the gene transfer, and (3) evaluating the endocytosis effect to analyze the biochemical changes. Based on the results, it was clear that RONS do not work alone for the process of gene transfection without electrical parameters such as electrical current, electrical charge and electrical field. According to the results of plasma induction on liposomes, under the condition without biochemical reactions, the process of plasma gene transfection occurs through endocytosis and electrical poration and not by the permeation through ion channels or chemical poration. A conceptual diagram of the factors affecting plasma gene transfection is depicted in Figure 4 [124].

Tissue engineering is considered a multi-phase technique composed of (a) isolating and extracting healthy cells from a patient, (b) in vitro incubation of cells in a bioreactor on a three-dimensional scaffold similar to the extracellular matrix (ECM), and (c) the transplantation of this 3D scaffold into the patient to replace the failing tissue until the newly generated tissue has matured to a particular degree [125,126]. The 3D scaffold substance must be both biocompatible (ideally bioactive) and degradable to function properly [127]. Most biopolymers are primarily employed in soft tissue applications because, from a mechanical standpoint, they are not suitable for load-bearing applications. The majority of synthetic material features are complementary to those of biopolymers with their benefits focusing on mechanical strength, availability, reproducibility, and pricing [128].

Surface chemistry and the presence of functional ligands should be taken into consideration when trying to emulate the natural extracellular matrix. Many proteins, including collagen, fibronectin, and others make up the ECM, which is a fibrous environment. Electrospinning (ES) was first patented in 1939, but it was not thoroughly investigated until the 1990s, when a resurgence of interest in one-dimensional scaffold manufacturing technology led to its development into the widely used tool it is today. ES can produce micro and nanofibers that replicate the structural characteristics of ECM [129,130]. Various scientific data revealed the efficacy of patterned surfaces on cell adhesion. Plasma-deposited patterned surfaces showed cell attachment even for human U937 macrophages, which ordinarily resist growing and attaching on tissue culture plates [131,132,133]. However, in order to address some serious concerns about the stability and aging of these kinds of thick scaffolds, Drews et al. [134] investigated thin plasma maleic anhydride coatings and identified that the thin films were more stable compared to the thick films [134,135]. 

Three-dimensional (3D) implants are also a revolutionary development in tissue engineering. Various research works are conducted with 3D implants to find a better way to modify and utilize the implants with plasma. Allylamine-based plasma polymerization in a polylactic acid (PLA) scaffold was considered one of the major improvements in this field. Allylamine plasma improved the cell adhesion property of PLA by penetrating nitrogen species to the inner surfaces virgin lactic acid polymer. In comparative in vivo studies in 3T3 fibroblast cells, Barry and co-workers proved that the cell adhesion properties of the plasma-modified PLA were much greater than those of the normal analog [136,137]. Choi et al. [138] demonstrated another surface modification study on calcium phosphate scaffolds using atmospheric pressure plasma jets with air and nitrogen gases [138]. The decrease in water contact angle from 80^0^ to 10^0^ was a proof of the improved hydrophilicity. XPS analysis showed that hydrophobicity was brought on by the substantial carbon contamination on the surface. This contamination was significantly decreased by the plasma treatment in favor of the addition of -OH groups to the surface [139]. 

The Plasma-Assisted Bio Extrusion System (PABS) is a pressure-assisted and screw-assisted plasma jet assembly that can improve the hydrophilicity of biomaterial scaffolds. Liu et al. [140] made a hybrid scaffold from a synthetic biopolymer and a natural hydrogel composed of alginate and gelatin. The nitrogen plasma-modified hybrid hydrogel of these materials showed a significant improvement in its surface hydrophilicity and thus by the cell proliferation. It is expected that the functionally regulated patterns will probably be robotically created on surfaces made of biomaterials [140,141].

## 3. Low-Temperature Plasma on Medicinal Implants

### 3.1. Low-Temperature Plasma-Based Orthopedic Implants 

**Figure 5:**
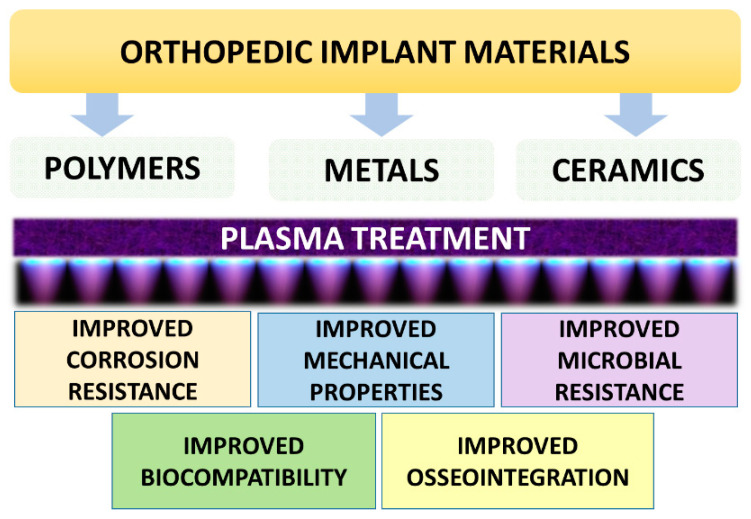
Types of orthopedic implants and plasma-induced properties for orthopedic implants.

In addition to other medical procedures, LTP can be used to prepare or enhance surfaces such that they are medically biocompatible, such as functionalizing implant surfaces. Orthopedic surgeons are particularly interested in the concept of osseointegration, which is the direct organizational adaptation of bone to the implant [142]. Surface modifications may improve the osseointegration of implants and can influence the success or failure of reconstructive surgery depending on the amount of bone anchoring on the implant surface [143]. Protein adsorption is regarded to be the first step in osteointegration, which then attracts osteoprogenitor cells to the implant surface. In order to create a favorable microenvironment on the implant surface, it is possible to manipulate the surface features of the implant such as charge, texture, and polarity to increase cellular adherence [144,145]. A material’s capacity to promote osseointegration is frequently assessed by looking at its surface energy, which is a measurement of unbonded surface atoms. Cellular adhesion is promoted by states with high surface energy [146,147]. As it has been proved that surfaces with a moderate degree of roughness outperform surfaces that have been turned, current research focuses on other modifications that could potentially boost the bioactivity of the implant. To induce coactive effects, some recent experiments have resorted to chemically altering somewhat abrasive surfaces [148]. A number of different materials are being used as orthopedic implants that can improve their properties by the application of LTP treatment (Figure 5).

Modified materials for hard tissue replacement are one of the major clinical demands at present. Individuals who receive orthopedic implants run the risk of developing implant-related infections (IAIs). Antibiotic-resistant bacteria are on the rise, which poses a challenge to IAI treatment [149]. Revision surgeries are more difficult, time-consuming, expensive, and subject to more risks than initial operations [150]. Many orthopedic implants and intramedullary rods are made of titanium as a common material. Several significant materials and methods were developed to improve the properties of such materials [151,152,153,154]. Many recent studies show that bacterial adhesion to plasma-treated titanium surfaces is comparatively low compared to stainless steel or normal titanium materials [155,156,157]. Wang et al. [158] examine the effect of low-temperature plasma on titanium-based implants to improve its surface biological functions. They have used low-temperature argon–oxygen plasma (LTAOP) as a plasma source to enhance the hydrophilicity of aged titanium surfaces. The analytical results showed that the plasma treatment makes the surface of the aged titanium materials more hydrophobic without any morphological changes. The LTAOP activation also improves the mineralization, proliferation, and adhesion of osteoblasts on the titanium surfaces [158]. Implants that were biofunctionalized by plasma electrolytic oxidation with Ag, Cu, and Zn exhibited considerable antibacterial action against a variety of bacteria, including ones that were resistant to antibiotics [159,160]. But metal implants raise a couple of serious health issues as well. Nickel–titanium (Ni-Ti) shape memory alloys are beneficial orthopedic biomaterials because of their extreme elasticity and form memory, although there are some safety concerns with the toxic nickel ions diffusing out of the body after continuous use [161,162]. 

A high-performance organic polymer called Poly Ether Ether Ketone (PEEK) has been created as a viable substitute for metallic implants. It is an interesting biomaterial with mechanical properties similar to those of human bones, excellent chemical resistance, and radiolucency [163,164,165,166]. Although polymers are interesting materials for many medical purposes, they are rarely utilized in their purest form. PEEK implantable materials would better adhere to the surrounding bone tissues; however, one of the main concerns associated with PEEK-based orthopedic implants is the slow appositional development of bone in vivo. To improve the orthopedic utility of PEEK, Wakelin et al. [167] employed an LTP technique, Plasma Immersion ion Implantation (PIII), which covalently immobilizes biomolecules to the material surfaces [167]. The PIII grafting on the surface of polymer implants also enhances the osseointegration and surface energy [168,169]. Along with these biomaterials, ceramics or ceramics-like materials also have been developed and utilized in modern implantology [170,171,172]. Modern implantology still heavily relies on hydroxyapatite-based osteoconductive and increasingly osteoinductive coatings [173]. Liu et al. [174] designed and synthesized a ceramic-like structure in situ on Polyamine 66 (PA66) by a plasma immersion ion implantation. The plasma treatment improved the surface hardness, cytocompatibility, and antibacterial capacity of this aminopolymeric implant [174]. Coatings made of CaO-SiO_2_ have also been approved as an attractive option for artificial implants with their superior bioactivity and biocompatibility but lacking mechanical strength under heavy load. Plasma-sprayed coatings of CaO-SiO_2_ can further increase the strength of the ceramic material. Similarly, plasma-sprayed wollastonite or plasma-sprayed dicalcium silicate coatings can have excellent bonding strength and biocompatibility [175,176,177,178]. 

### 3.2. Low-Temperature Plasma: Application in Dentistry

Dental caries (tooth decay) and gingival inflammations periodontal diseases (inflammation of supporting dental tissues) are the most common dental diseases caused by microorganisms, which can be treated at the early stages when it shows the preliminary indication of tooth discoloration. However, negligence of this may lead to severe periodontal inflammation that leads to the eventual loss of the tooth. The use of laser devices and the application of antimicrobial solutions are the two standard procedures for oral cavity disinfection. However, these conventional procedures might result in thermal/mechanical damage to the healthy periodontal tissues because of the use of ozone, hydroperoxides, mechanical drilling, etc. in the treatment methods [179]. In this scenario, LTP-based techniques emerged as an ideal candidate for treating various dental and related diseases, which can operate at room temperature with minimal tissue damage. The applications of low-temperature plasma have been effectively used in the sterilization of dental devices, dental composite/adhesive restoration, safe destruction of dental biofilms and plaques, root canal disinfection, dental whitening, etc. 

The short-lived chemical species from plasma devices were subjected to quite a lot of dental research to decimate the biofilms (dental plaques) caused by cariogenic microorganisms such as *Streptococcus mutans* [180], *Basillus cereus*, *Geobacillus stearothermophilus* [181], *Lactobacillus acidophilus* [182], *Phophyromonas gingivalis* [183], *Lactobacillus casei*, *Candida albicans* [184], etc. As an in vitro proof for the antimicrobial efficacy of NPT against dental biofilms, Koban and co-workers carried out a comparative study that assessed the efficiency of three different NTP devices against the biofilms created by *Streptococcus mutans* and saliva multispecies grown on titanium discs and compared its efficiency with chlorohexidine, which is an anti-inflammatory solution used to treat gingivitis. The results revealed that the efficacy of NTP device-induced dental plaque destruction is almost two-fold higher than that of chlorohexidine [185]. In endodontic treatment, one of the difficult tasks is to achieve the complete sterilization of infected root canals (RCs). Traditional treatments like mechanical debridement, chemical irrigation, laser irradiation, or ultrasound techniques were unable to attain maximum disinfection because of the complexity of the RC systems. To address this difficulty in the RC procedure, Pan et al. [186] studied the effect of LTP in root canals infected with the bacteria *Enterococcus faecalis*. Their analysis of the microscopic images of the bacterial biofilms before and after the plasma treatments point out that the plasma treatment has high efficiency in rupturing the *E. faecalis* biofilms in the RC treatment [186]. 

**Figure 6:**
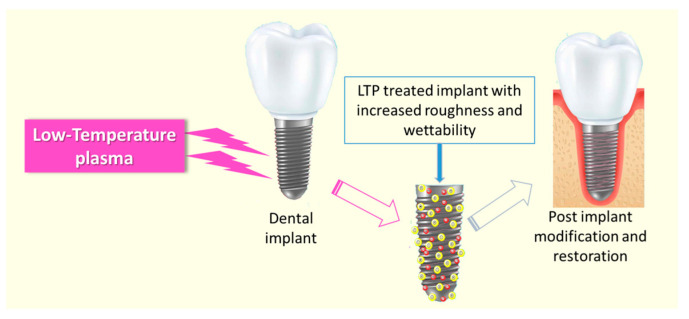
Schematic representation of the LTP-based dental implant modification. Adapted and modified from Lata et al. Aurora Borealis in dentistry: The applications of cold plasma in biomedicine. Mater. Today Bio 2022, 13, 100200. [187] with copyright permission from Elsevier.

LTP has also been used in the surface modification of dental implants to increase the surface roughness and wettability of the implant material (Figure 6) [187]. The major advantage of the plasma-treated implant is that it can favor cell adhesion and restoration without any residual surface-free energy or chemical impurities [188,189]. It was well established that the non-thermal plasma treatment demineralizes dentin, which improves the adhesive–dentin bonding strength [190,191]. Many researchers independently studied to understand the mechanism of the improved adhesive–dentin interface bonding [192,193,194]. The morphological analysis of the plasma-modified dentin showed that the plasma induction collapses the collagen helix structures of the dentin surface, which helps the penetration of monomers/resin into the dentin tubules and the collagen network. Being a great source of RONS and the advantages of non-thermal characteristics, LTP-based tooth bleaching has received considerable attention in dental research. It was reported that the combined application of LTP with modern bleaching agents such as hydrogen peroxide [195] and carbamide peroxide [196] considerably increases the anti-staining efficiency. The intracoronal tooth bleaching using LTP-assisted 30% hydrogen peroxide was more effective than that with H_2_O_2_ alone, and the induction time of plasma has a significant effect on the efficiency of the process [195].

## 4. Low-Temperature Plasma-Assisted Biomaterials Process in Alabama

With the grant support of tens of millions of dollars ($40 million) for research and infrastructure programs under the titles ‘Connecting the Plasma Universe to Plasma Technology in Alabama (CPU2AL)’ and ‘Future Technologies and Enabling Plasma Processes (FTPP)’ from U.S National Science Foundation (NSF), the plasma research is progressing in Alabama. These two programmatic projects seek to understand, predict, and control plasma processes and interactions in LTP environments to develop new technologies for aerospace, manufacturing, medicine, agriculture, and food safety. These projects comprised a consortium of Alabama universities and industries with each partner institution possessing strengths individually in theory, modeling, experiment, and/or plasma technologies. At the University of Alabama at Birmingham (UAB), Thomas’ group focuses on ‘plasma interactions with biomaterials and soft-matter’ with research thrust in surface engineering of polymers and biomaterials for cardiovascular and bone applications. New plasma methods such as cold plasma processing for modifying the intimal surface of small caliber tubular conduits [197] and magneto-plasma processing for accelerating the surface modifications [198] were reported. Poly (tetrafluro ethane), PTFE, is a non-sticky polymer, and expanded PTFE form is a widely known fibrous material for stable-vascular graft application, which is limited to large or medium diameter graft applications. Recently, our research group employed organic plasma processing (OPP) methods to polymerize monomers of methyl methacrylate (MMA) or silane using RF plasma techniques to coat PTFE surfaces [199,200]. The new hybrid mix of oxygen–MMA plasma produced a superhydrophobic Cassie–Baxter state surface with a 154° water contact angle. The surface etching of PTFE by oxygen plasma and the following surface chemical modification/deposition mediated by O_2_/MMA plasma can account for the observed super hydrophobicity phenomenon. Water droplets will roll off the surfaces due to the strong water repellency, clearing the surface of any potential pollutants or impurities (self-cleaning). We believe there are various possible bio-interface applications worthy of further research, and this new combination of top–down (plasma etching) and bottom–up (chemical vapor modification) offers an upgrade in the design of superhydrophobic PTFE surfaces. Contrary to the superhydrophobic surface, we designed a novel and facile organosilane plasma polymerization method to improve the hydrophilicity of PTFE for blood contact interfaces [200]. The plasma polymerization of silane could augment the PTFE surface properties with characteristics such as high hydrophilicity, high surface oxygen content, improved endothelial cell adhesion, and reduced platelet adhesion for vascular graft applications.

Recently, we reported the use of dusty plasma for generating nanoparticles onto 3D-printed tissue bone scaffolds [201]. These nanoparticles were formed in situ and anchored onto the biomaterial surfaces. Antibacterial plasmonic nanoparticles of silver and gold could also be prepared by a process called ‘Plasma Electroless Reduction’ (PER) [202]. These metallic nanostructures demonstrated antibacterial competence against both Gram-positive and Gram-negative pathogenic bacteria, particularly the silver nanoparticles (AgNp) coating on surgical mask layers or 3D-printed polymer scaffolds. We believe that the PER technique will be very helpful in the designing of metallic nanostructured interfaces for many biomedical uses, including bone tissue engineering and biosensing [203]. Moreover, our group recently published two invited mini-reviews on mitigation strategies in engineered healthcare materials toward antimicrobial applications and non-thermal plasma processing for nanostructured biomaterial [204,205] in the journal named *Current Opinion in Biomedical Engineering*. Plasma research and technologies will establish Alabama as a southeastern regional hub for plasma science expertise and create thousands of high-paying technical careers in the state and region.

## 5. Conclusions

Cold atmospheric plasma or low-temperature plasma-based biomedical applications and therapeutics have emerged in the last two decades and addressed several long-standing biomedical complexities. The invention of the interaction of plasma with soft materials like biological cells and tissues was a breakthrough in the applications of plasma in modern medicine; however, the LTP–cell interactions still lack a suitable mechanistic explanation. This review presents some of the potential developments in LTP-based therapeutics and biomedical applications including its efficiency in antimicrobial therapy, cancer treatment, drug/gene delivery, orthopedic implants, dental treatments, interaction with biomolecules, etc. In the current scenario, LTP technologies have to address several challenges including (1) the safety of LTP sources that use a power supply to initiate and propagate plasma, (2) technical advancements in the complex LTP’s diagnostics and simulations, (3) several fundamental questions and the mechanistic understanding of plasma interaction on tissues and organisms, (4) a clear mechanism for the synergic anti-cancer effect of LTP or plasma-activated liquids (PALs), etc. Studies should go beyond in vitro experiments to prove the medicinal applications of low-temperature plasma. In addition to the in vivo experiments and clinical trials, research works might include the possibilities of artificial intelligence (AI) and customized electronic sensors for the further advancement of the field of plasma medicine.

## Data Availability

Not applicable.
